# Community health workers of Afghanistan: a qualitative study of a national program

**DOI:** 10.1186/1752-1505-8-26

**Published:** 2014-12-01

**Authors:** Said Ahmad Maisam Najafizada, Ronald Labonté, Ivy Lynn Bourgeault

**Affiliations:** Population Health Program & Institute of Population Health, University of Ottawa, 1 Stewart Street, Ottawa, ON K1N6N5 Canada; Department of Epidemiology and Community Medicine, Faculty of Medicine & Institute of Population Health, University of Ottawa, Ottawa, Canada; Telfer School of Management & Institute of Population Health, University of Ottawa, Ottawa, Canada

**Keywords:** Community health workers, Afghanistan, Post-conflict countries, Rural health workforce, Health system strengthening

## Abstract

**Background:**

Afghanistan is a country that has been in conflict for decades, resulting in the destruction of much of its social infrastructure including the health system. In 2003, after the intervention of US-led NATO forces, the new government with support from its international partners designed a Basic Package of Health Services to provide services to the majority rural population; its specific focus is on women and children. The workforce to deliver these services consists of Community Health Workers (CHWs). In this paper we aim to 1) describe the CHW program, 2) explore the gender dynamics of the workforce, and 3) identify facilitators and challenges to the program.

**Method:**

Our descriptive, qualitative study involved an analysis of policy and administrative documents, in-depth interviews and focus groups, and non-participant observation. Ethical approval for the fieldwork was obtained from the University of Ottawa, and the Afghanistan National Public Health Institute.

**Results:**

There are more than 20,000 CHWs across the country serving as village primary care providers, functioning as a liaison between the community and health-care facilities, and working as community developers; more than half are women. Noteworthy is a gender hierarchy: as one moves up the hierarchy of supervision and training, management and decision-making, the ratio of women to men diminishes. We found that female CHWs accomplished their tasks vis-à-vis maternal child health with greater ease than their male counterparts, as societal gender dynamics influences task allocation. Volunteerism helps to deploy a larger number of CHWs, but also makes their retention difficult. Community participation facilitates tasks of CHWs, but also poses challenges to the program, such as traditional leaders influencing the recruitment of CHWs that may not be the best choice for the community. Drug supply and support for CHWs is vital to the effectiveness of the program.

**Conclusions:**

This case study of the decade-long, rural health workforce CHW program in Afghanistan suggests that CHWs play an important role in post-conflict, developing countries, potentially contributing to health system strengthening.

## Background

Armed conflicts leave behind devastating health and social system impacts [1]. The detrimental effects of conflict are particularly long lasting in low-income countries because of the consequent slow reconstruction of infrastructure [2]. One of the major obstacles to establishing a health system in post-conflict and fragile states is a shortage of health human resources [3]. Community Health Workers (CHWs) have been deployed in various countries to help address these shortfalls [4]. CHWs are most often community members who are trained, supported and supervised by more formal health professionals to deliver primary health services to their communities [4]. Examples of such workers integrated into national health systems have been documented in Ethiopia, Pakistan, India, Iran and Brazil [5–9]. Afghanistan has recently deployed CHWs as a part of its national health care services.

Afghanistan is a multiethnic and multilingual country of approximately 30 m people [10]. Afghanistan’s Human Development Index (HDI) is one of the lowest in the world, in particular for social indicators pertaining to women. Only 6 percent of women in Afghanistan have at least secondary education, and their labour participation rate is 16 percent [11]. The culture of *purdah* (gender segregation) is practiced all over the country at differing levels, depending on various ethnicities, and with rural–urban differences [12]. In the 1990s, during the civil war and the time of the Taliban, women were barred from social, economic, and political lives, and restricted to their homes.

Afghanistan has also been in conflict for decades. This has included the war against Russian invasion, the civil war, and most recently the war on terrorism and insurgency [13]. The civil war of the 1990s destroyed much of the social infrastructure of the country including the health system [14]. In 2003, after the intervention of US-led NATO forces, the new government with support from its international partners and government and non-government organizations designed a Basic Package of Health Services (BPHS) to provide services to the majority rural population; its specific focus is on women and children [15].

BPHS is the foundation of the public health care services in Afghanistan. Delivery of the package is principally contracted out to national and international NGOs due to lack of government capacity to provide the services. Three provinces in close proximity to Kabul also implement the package as part of a process to build the capacity of public organizations to provide the services, referred to as the Strengthening Mechanism (SM) or contracting-in [15].

The health workforce to deliver the services at the rural level is comprised of Community Health Workers (CHWs) [15]. CHWs in Afghanistan are local volunteer community members, approved by a community council, and trained, supported and supervised by a health organization [15]. Today, there are more than 20,000 CHWs across the country serving village primary care providers, functioning as a liaison between the community and health-care facilities, and working as community developers [16]. More than half of CHWs are women [16].

In this paper, we 1) describe the CHW program, 2) explore the gender dynamics of the workforce, and 3) identify facilitators and challenges to the program.

## Method

A descriptive, exploratory qualitative design was chosen to examine the CHW program in post-conflict Afghanistan. Exploratory studies can provide insight into a program, revealing contextual factors affecting the program that may not have been taken into account when the program was being designed or implemented [17, 18]. The data for this study included policy and administrative documents, in-depth interviews with policy makers and health managers, and in-depth interviews and focus groups with CHWs and community members.

Documents were collected from the Afghanistan’s Ministry of Public Health (MoPH) and international donors and partners: United States Agency for International Development (USAID), the World Bank, the European Union/Commission (EU), World Health Organization (WHO), Department of Foreign Affairs, Trade and Development Canada (DFATD), United Nations Population Fund (UNFPA). Documents were also collected from implementing organizations: international Non-Governmental Organizations (NGOs), national NGOs, and local provincial health departments. We source publically available documents from these organizations and their websites, and documents not publically available through personal contacts with those organizations.

**Figure 1:**
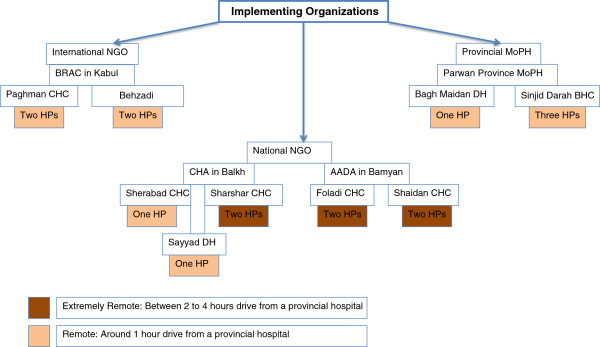
**Three types of implementing organizations and the sampling diagram.**

A mix of purposive and convenience sampling methods were used for the in-depth interviews. Policymakers from the MoPH, aid agencies, and UN organizations were selected based on their knowledge of the BPHS. Health managers were selected based on the three types of service providers in provinces (International NGOs, national NGOs, and provincial government public health departments). Selection criteria for provinces and the villages were security (*of the field researcher*), distance from a provincial hospital, ethnicity of the village population, gender of CHWs (1 male and 1 female), and availability of CHWs. We selected two national NGOs for one of them had remote health posts meeting the criteria. Selection criteria for focus group participants were available male and female community members who had received services of CHWs or were otherwise involved in the CHW program. Figure 1 illustrates the sampling diagram and the number of HP.

The lead author [MN] and his assistant took field notes during and right after the field visits to document their observations. They spent between 15 minutes to 1 hour at each health facility that covered the selected villages, observing the health facility, its wards, patients, and services provided for the patients, and visiting health care providers in the health facility. They also spent between 30 minutes to 1 hour at each village identifying sources of drinking water and the location and the type of latrines, and chatting with villagers on health services. The field notes included a journal of what happened during these visits, relevant statements, and the two researchers’ initial analytical reflections. The observation notes were used to crosscheck or complement the interview data, and to assist in data analysis.

A total of 25 CHWs, 9 CHW supervisors, 4 CHW trainers, 6 health facility and implementing organization managers, and 11 policy makers were interviewed. Eight focus groups with community members were conducted between 5 July and 30 September 2013. The participants at the policy and managerial levels were from the MoPH, WHO, DFATD, UNFPA, EU, Bangladesh Rural Advancement Committee (BRAC), Coordination for Humanitarian Assistance, Agency for Assistance and Development of Afghanistan, and provincial health department of Parwan. Interviews with policy makers and health managers took place at their office at their availability. Interviews with CHWs and focus groups with community members took place at HPs, except in one district where the interviews took place at the health center for cultural reasons. The interviews lasted between 30 to 90 minutes. All interviews and focus groups were semi-structured. The number of focus group participants varied between 3 and 7 people. They were male-only and/or female-only focus groups, except in three villages in Bamyan where both male and female community members participated in the same focus groups.

Ethical approval for the fieldwork was obtained from the University of Ottawa (#H05-13-07), and the Afghanistan National Public Health Institute (#356377). Written consent forms were signed or oral consents were recorded. For written consent, participants were given time to read the consent forms and ask questions before signing. For oral consent, the objectives of the research, and ethical issues related to safety, confidentiality, and privacy were explained to them in the local language before asking if agreed to the interview or focus group. All of the participants agreed to digital recording; and most of them agreed to being photographed, except for 2 female CHWs and 10 female community members who did not allow to be photographed for cultural reasons.

The audio-recorded interviews and focus groups were translated and transcribed simultaneously by the lead author. The final transcripts were treated as the data for analysis. Initial analysis of the interviews began during the fieldwork, when the lead author wrote two fieldwork reports to his supervisors. Final thematic analysis was carried out through reading and re-reading the compiled transcripts. Manual coding [17] of the transcripts was the first step towards data reduction. Patterns within the codes were summarized as sub-themes and displayed in the form of a matrix. Constant comparison technique [17, 18] was used to evaluate how emerging sub-themes correlated or differed with each other. Finally, themes emerging from the similar sets of sub-themes were derived [17, 18]. Confidentiality was practiced at every stage including anonymising the quotes from the interviews for this paper.

## Results

Demographic data on our key informants are provided in Tables 1, 2, 3, 4, 5, 6, 7, 8. Table 1 and Table 2 shows the number of all key informants at different levels, and their gender respectively. Table 3 provides demographic information on the villages where CHWs operated. Table 4 is a demographic presentation of CHWs, a majority of whom are women, and 25% of whom have not received the basic training. Table 5 provides similar data for CHS who, collectively, supervise 255 CHWs in 128 HPs. Tables 6, 7, 8 describe CHW-trainers, health managers and policymakers. Noteworthy from these tables is a gender hierarchy; that as one moves up the hierarchy of supervision and training, management and decision-making, the ratio of women to men diminishes. We discuss this later.

**Table 1:** **Interview participants**

Actual interview participants			
	Participants	Participants’ criteria	Number
Policy Makers	Ministry of Public Health	Involvement in CHWs program design and implementation (Health Officers, Health Advisors, Community-based Health Care Department officers, Health Economics and Finance Department Consultants, Deputy Minister of Policy and Human Resources)	4
	USAID		1
	World Bank		1
	European Commission		2
	DFATD - Canada		1
	WHO		1
	UNFPA		1
		Sub-total	11
Health service organizations	Health Managers	International NGO	6
	CHSs	National NGO	9
	CHW Trainers	Provincial Health Department	4
		Sub-total	19
Community	CHWs		25
	Community members		25
		Sub-total	50
		**Total**	**80**

**Table 2:** **Gender of key informants**

Key informants	Male (%)	Female (%)	Total
**CHWs**	9 (36%)	16 (64%)	25
**Supervisor & Trainer**	9 (69%)	4 (31%)	13
**Health managers**	6 (100%)	0 (0%)	6
**Policy makers**	9 (82%)	2 (18%)	11

**Table 3:** **Catchment area/villages**

Health post/Village name	# of CHW	CHW gender ratio	District	Province	Health facility	Household	Persons	Village ethnicity	Distance from clinic	Drinking water	Electricity
Zakria Foladi	2	1–1	Central Bamyan	Bamyan	CHC Foladi	75	549	Hazara	2 hour walk	Yes	Yes
Kata Sang Foladi	2	1–1	Central Bamyan	Bamyan	CHC Foladi	150	1100	Hazara	1 hour walk	Yes	Yes
Naal Sheran	2	Two female	Central Bamyan	Bamyan	CHC Shaidan	102	840	Hazara	1 hour walk	No	Yes
Habashi	2	Two female	Central Bamyan	Bamyan	CHC Shaidan	155	930	Hazara	2.5 hour walk	No	Yes
Deh-Yaqub	2	1–1	Behzadi	Kabul	BHC Behzadi	110	770	Tajik-Pashtun	2 hour walk	No	Yes
Qala-e Jan Baz	2	Two female	Behzadi	Kabul	BHC Behzadi	120	780	Tajik-Pashtun	1.5 hour walk	No	Yes
Qala-e Sarwar	2	1–1	Paghman	Kabul	CHC Paghman	120	1140	Pashtun	1.5 hour walk	Yes	Yes
Poshta-e Badam	2	1–1	Paghman	Kabul	CHC Paghman	300	2100	Pashtun	1 hour walk	Yes	Yes
Khalazaye	2	1–1	Charikar	Parwan	BHC Sinjid Dara	1000	5000+	Pashtun	2 hour walk	No	No
Deh Rayes	2	1–1	Sinjid Darah	Parwan	BHC Sinjid Dara	150	1050	Tajik	1 hour walk	Yes	Yes
Deh Neshar	2	1–1	Sinjid Darah	Parwan	BHC Sinjid Dara	320	2240	Tajik	1.5 hour walk	No	Yes
Nawoch Sufla	2	1–1	Salang	Parwan	DH Bagh Maidan	170	1190	Tajik	0.5 hour walk	Yes	Yes
Sayyad	2	Two female	Kholm	Balkh	DH Kholm	400	2900	Tajik	2 hour walk	No	Yes
Abdul Hamid	2	Two female	Dehdadi	Balkh	CHC Sherabad	160	1120	Tajik	1 hour walk	Yes	Yes
Shanjeer	2	1–1	Charkent	Balkh	CHC Sharshar	263	1841	Hazara	3 hour walk	Yes	No
Karmagali	2	1–1	Charket	Balkh	CHC Sharshar	100	700	Hazara	4 hour walk	Yes	No

**Table 4:** **Community health workers**

Number	Title	Age	Gender	Marital status	Education	Experience in years	Other occupation	Province	Basic training
1	**CHW**	17	F	Single	10th Grade	4	Student	Bamyan	No
2	**CHW**	18	M	Single	12th Grade	1	Student	Bamyan	No
3	**CHW**	21	F	Single	Teacher Training	11	School Teacher/Veternarian	Bamyan	Yes
4	**CHW**	19	M	Single	11th Grade	2	Student	Bamyan	No
5	**CHW**	28	F	Married	12th Grade	4	Teacher	Bamyan	No
6	**CHW**	32	F	Married	9th Grade	6	Teacher	Bamyan	No
7	**CHW**	40	F	Widow	Illiterate	6	Dai*	Bamyan	Yes
8	**CHW**	45	F	Married	Illiterate	7	Dai*	Bamyan	Yes
9	**CHW**	41	F	Married	12th Grade	1.2	Teacher	Kabul	Yes
10	**CHW**	24	M	Single	BA	10	BA in political science	Kabul	Yes
11	**CHW**	18	F	Single	6th Grade	5	Student	Kabul	Yes
12	**CHW**	40	F	Married	Illiterate	5	Housewife	Kabul	Yes
13	**CHW**	40	F	Married	Illiterate	10	Housewife	Kabul	Yes
14	**CHW**	22	M	Single	12th Grade	7	Teacher	Parwan	Yes
15	**CHW**	50	F	Widow	Illiterate	7	Dai	Parwan	Yes
16	**CHW**	55	M	Married	12th Grade	6	Cashier	Parwan	Yes
17	**CHW**	35	M	Married	12th Grade	5	School Headmaster/Teacher	Parwan	Yes
18	**CHW**	35	M	Married	Illiterate	5	Water Distributer	Parwan	Yes
19	**CHW**	69	F	Widow	Illiterate	9	Dai	Balkh	Yes
20	**CHW**	37	F	Married	12th Grade	10	Tailor	Balkh	Yes
21	**CHW**	27	F	Married	8th Grade	10	Tailor	Balkh	Yes
22	**CHW**	47	M	Married	Illiterate	5	Farmer/Religious Services	Balkh	Yes
23	**CHW**	45	M	Married	Religions studies	11	Community worker	Balkh	Yes
24	**CHW**	40	F	Married	Illiterate	5	Housewife	Balkh	Yes
25	**CHW**	40	F	Married	Illiterate	5	Housewife	Balkh	No

*Dai: Traditional birth attendant.

**Table 5:** **Community health supervisors**

Number	Title	Age	Gender	Marital status	Education	Experience/(years)	Province	# of health posts	# of CHWs	# of Male CHWs	# of female CHWs
1	CHS	37	M	Married	12th Grade	3.5	Bamyan	27	54	18	36
2	CHS	22	M	Single	12th Grade	0.5	Bamyan	19	38	16	22
3	CHS	50	F	Married	9th Grade	1.5	Kabul	8	16	3	13
4	CHS	42	M	Married	12th Grade	4	Kabul	17	34	12	22
5	CHS	45	M	Married	12th Grade	2	Parwan	9	18	14	4
6	CHS	35	M	Married	12th Grade	3	Parwan	14	28	18	10
7	CHS	45	M	Married	12th Grade	8	Balkh	10	20	12	8
8	CHS	35	F	Married	12th Grade	10	Balkh	15	29	13	16
9	CHS	30	M	Married	12th Grade	3	Balkh	9	18	13	5

**Table 6:** **Community health worker – trainers**

Numbers	Occupation	Age	Gender	Marital status	Education	Experience (years)
1	CHW Trainer	35	F	Married	12th Grade	7 years
2	CHW Trainer	55	M	Married	Military School	4 years
3	CHW Trainer	35	F	Married	BA	5 years
4	CHW Trainer	40	M	Married	Pharmacist	2 years

**Table 7:** **Health managers**

Numbers	Occupation	Age	Gender	Marital status	Education	Experience (years)
1	Program Manager	35	M	Married	MD	5 years
2	Hospital Manager	50	M	Married	MD	7 years
3	Health Advisor	30	M	Married	MA	3 years
4	CBHC Officer	50	M	Married	BSc in Health Sciences	10 years
5	Program Manager	55	M	Married	MD	9 years
6	CBHC Officer	32	M	Married	MD	2 years

**Table 8:** **Policymakers**

Number	Organization	Age	Gender	Education	Department
1	MoPH	55	M	MD/MPH	Community-Based Health Care
2	MoPH - Int Org	45	M	MD	Grant and Contract Management Unit
3	MoPH - Int Org	41	M	MD	Grant and Contract Management Unit
4	MoPH - Int Org	42	M	MD	Grant and Contract Management Unit
5	MoPH	34	M	MD	Health Economics and Finance Department
6	MoPH	27	F	MA	Health Economics and Finance Department
7	MoPH	56	M	MD	Professional Affairs Department
8	Int Org	36	F	MA	Health & Development
9	Int Org	40	M	MD/MPH	Gender and Community-Based Services
10	Int Org	42	M	MD/MPH	Community-Based Services
11	Int Org	54	M	MD	Health & Development

### CHW program

**Figure 2:**
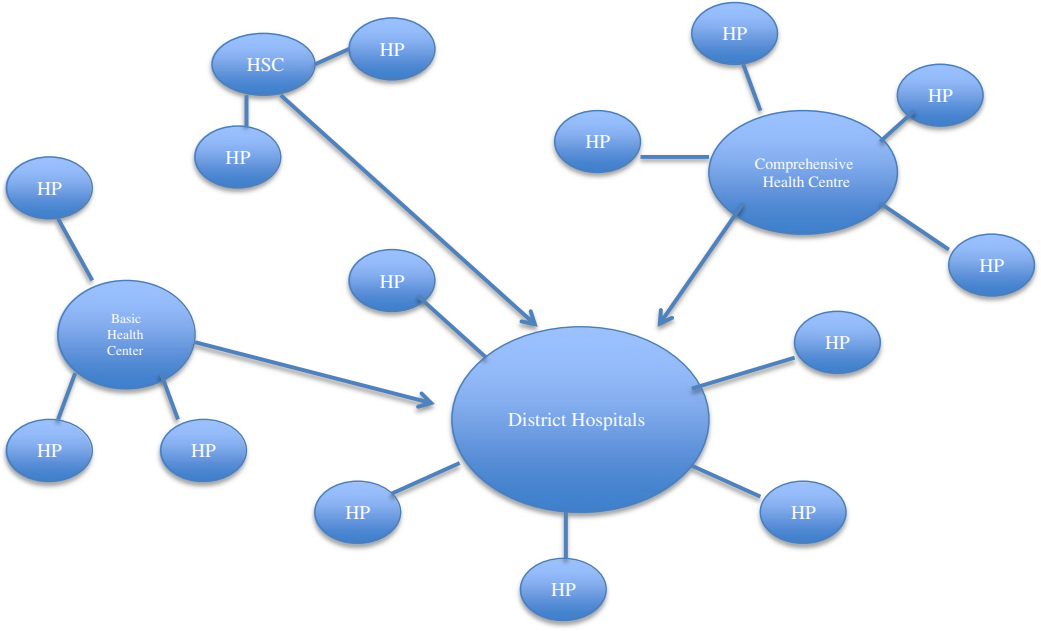
**The structure of the BPHS in rural Afghanistan.**

The CHW Program is a part of the Basic Package of Health Services, which in turn is the foundation of the public health care services in Afghanistan. The package has a semi-hierarchal structure with a Health Post (HP) at the bottom, and a District Hospital (DH) at the top, designed to cover a specific range of population, also called a catchment area. A HP (Catchment Area: 1,000-1,500 population) is a room in a villager’s house where typically one male and one female CHW are based. Facilities above the HP are a Health Sub-Center (HSC; CA: 3,000–7,000), followed by a Basic Health Centre (BHC; CA: 15,000–30,000), a Comprehensive Health Center (CHC; CA: 30,000–60,000), and then the District Hospital (DH; CA: 100,000–300,000) [15].Mapping catchment area in the villages has been tricky. Mostly, a village has become a catchment area, but small villages and large villages have made that mapping a complicated issue. All establishments above a HP are identified as Health Facilities (HF). The HP is the initial point of contact for the rural population with the health system. Every HF has a Community Health Supervisor (CHS), a staff responsible for supervision, support, and refresher training of CHWs. The relationships between HP and health facilities are not rigidly hierarchal. Not all HSCs are linked to a BHC, which in turn should be linked to a CHC, and then to a DH. HSCs can be linked to a CHC or directly to a DH (Figure 2).

### Community Health Workers & their tasks

CHWs are largely volunteer members of the community, nominated by a Village Health Council (VHC), and trained, supervised and supported by the organization implementing the BPHS. They are reimbursed for their trip to health facilities, and provided toothbrush and toothpaste, hand soap, and towel for their own use. Most CHWs are illiterate; few of them can read and write.

In Table 9, we compare the tasks designed in the BPHS with the ones CHWs actually undertake. For example, female CHWs participate in female-only events and meetings. CHWs participate in national campaigns only when they are asked or employed to do so. Mental health and tuberculosis carry stigmas, and thus are rarely reported or dealt with by CHWs, leading to their claim not to have many cases of those illnesses. We tentatively conclude that, based on our sample to date, active CHWs undertake most of the tasks they have been assigned in varying degrees, and only when the social/cultural context allows.

**Table 9:** **Comparison of tasks of CHWs in their job description and the ones they actually undertake**

Tasks in the job description of CHWs (BPHS, 2010)	If and how CHWs undertake the tasks
**Community collaboration and health promotion**	
1. Actively Participate in community meetings and events	Segregated by gender
2. Actively work with mother’s groups and Family Health Action Groups	Mother’s groups and FHA often do not exist
3. Encourage the community to participate in immunization	Yes
4. Participate in immunization campaigns	If asked by the campaign managers
5. Promote good nutrition practice	Yes
6. Promote the use of ORS and ZINC, and homemade rehydration	Yes
7. Promote hygiene and sanitation	Yes
8. Encourage couples to practice birth-spacing	Yes
9. Promote psychosocial and mental health in the community	No
10. Raise awareness on addictive substance	No
**Direct services**	
1. Treat mild prevalent diseases	Yes
2. Implement community-based IMCI	Yes
3. Implement community-based growth promotion with FHA groups	FHA groups often do not exist
4. Counsel on correct use of medications	Yes
5. TB prevention program	Rarely, due to rare cases of TB
6. Promote ANC and PNC	Yes
7. Encourage skilled birth attendance (and institutional delivery)	Yes
8. Distribute contraceptives	Yes
9. Provide first-aid services to the community	Yes
10. Ensure administration of Vit A to children	Yes
**Management**	
1. Meeting with community health council	Yes
2. Meeting with CHS	Yes
3. Support community midwives	Yes, if there is a midwife
4. Complete and submit Tally Sheet	Yes
5. Know the community and develop a community map	Yes
6. Report mortality and disease outbreak	Yes
7. Manage the HP, maintain supplies and drugs, and report utilization	Yes

In collaboration with village elder/leader and village council, CHWs also form a Village Health Council (VHC). These VHCs are male only, female only, or of mixed-sex, with one VHC in each village or catchment area. CHWs convene meetings of VHCs on a monthly basis, in which they discuss health issues of the village. Male only VHCs often discuss environmental health issues such as water for drinking and for irrigation, electricity, and roads for quick and easy access to the health facilities. Female only VHCs generally focus on maternal and child health such as breastfeeding, a good source of nutrients for mothers and children, antenatal and postnatal care visits, family problems (convincing mother-in-laws and husbands to allow them to access health care services), and provision of vehicles for transportation of pregnant women to the health facilities.

CHWs also attend a monthly refresher training/meeting at the HF to refresh their knowledge, and to discuss village health problems at the facility. Most CHWs attend the monthly meetings for a number of reasons. First, they are paid a travel stipend, but they usually walk the trip between the village and the health clinic for lack of transportation and save the money. Second, the monthly meeting refreshes the relationship of the CHW with the health facility, which in turn boosts the status of the CHW in the community on their return.

"…*because people think we might have brought* [*back*] *drugs or something to give them like brochures or posters*". (Male CHW)

Third, CHWs refresh their knowledge of the services they provide and learn new information. Finally, for female CHWs, going to clinic gives them a sense of freedom and empowerment.

"*Women do not go outside without their men*’*s permission*, *it is like taking a break*, *going to clinic*". (Female CHW)

#### Conversations about contraception

One of the important tasks of the CHWs is to distribute contraceptives and encourage people to use them. Contraceptives are used to reduce the number of children, and to increase the space between children. The latter is more common among younger women and the former among women in their late 30s. Female CHWs encourage women with four to five children to stop having babies; and women in their twenties to use contraceptives and to give two to three years of space between each pregnancy. The decision to use contraceptives is usually made by the head of the family.

Convincing community members to use contraceptives is a difficult task that female CHWs learn by practice. In some places, where gender segregation is not strictly practiced, female CHWs involve husbands in counseling sessions to make them aware of the risks of too many or too quickly spaced pregnancies. Female CHWs have learned that husbands’ involvement in the counseling sessions can be effective.

"…*when they say that their men* [*Husbands*] *do not allow them to use contraceptives*… *next time I go to their house at a time that her man is at home*, *and I ask her to call her man too*, *and counsel them together*". (Female CHW)

The decision to use contraception also depends on the number of children in the family, and whether the family has the desired number of boys. Normally, families want more children, and preferably boys. In response to how many children a family wishes to have, one CHW noted:

"*Mostly like four kids*, *boys and girls*, *but unfortunately when they do not have baby boys*, *they keep trying*". (Female CHW)

Sometimes women who wish to give space between their pregnancies but their in-laws want more children quickly take the contraceptives in secret, which creates tension between the family and the CHWs. CHWs are not expected to provide contraceptives to women without the approval of their family.

"…*her mother*-*in*-*law came here* [*at the HPs*, *saying*], ‘*you are doing bad*, *you are not allowed to give my daughter*-*in*-*law contraceptives*’… *she told the entire village that I had given contraceptives to her daughter*-*in*-*law secretly*… *that fight lasted two*-*three months*". (Female CHW)

### Selection and recruitment

To recruit a CHW, the CHS informs the community and encourages potential candidates (female, somewhat literate, willing to help her fellow villagers) to become a CHW. Although there is no minimum literacy level required to become a CHW, there are many illiterate CHWs who are undertaking their tasks with success. The minimum criteria for selecting a CHW, albeit not explicit in the written policy, are the agreement of a community member to work as a volunteer and to turn a room in his/her house into a health post. Once that person agrees to become a CHW, a village council (typically comprised of traditional leaders) approves his/her decision, and the person is introduced to the HF to receive training.

"*Local leaders like elders*, *teachers and Mullahs have a role in recruitment of CHWs*". (Health Manager)

Local leaders have two advantages. First, they are community gatekeepers who can become a change agent for healthy behavior and environment. Second, they usually have a guestroom, which can be turned into a health post.

Turning one’s room into a HP means putting health-related posters on the walls of the room, and keeping the health kit (a box of drugs, dressing materials, condoms, pills, injections, and other health-related materials) in the room to provide to the villagers when they need, and holding meeting of VHCs. The villagers come to the HP for drugs, contraceptives, health advice, pregnancy-related matters, mild injuries, and sometimes injections. It becomes a care center for patients and the community, but rarely is it used for overnight patient stay.

### Training

The candidates are trained by the organization contracted to implement the CHW/BHPS program, which includes a standard training module. Training consists of three rounds of 18-days with 2 months of fieldwork in between each of the trainings. CHWs are provided with a pictographic training manual (easy for those who are unable to read), which includes a range of public health awareness and education tasks, a number of direct services such as family planning, nutrition, maternal and child health, and treatment of common diseases, such as diarrhoea and pneumonia. After the first 18-day training, the candidate is identified as an active CHW and will start serving the population. After completing the third round of 18-day training, the CHW will receive a certificate from the Ministry of Public Health and the implementing organization. The CHWs will receive 1 refresher training every 6 months. The refresher training is for three-days [19].

Training is an essential component of program success. CHWs recruited at the beginning of the BPHS program in 2003 and 2004, received proper basic training for 6 months (three 18-day in-class and 4 months field work). They had not learned much since then. Most of them asked for more training on issues outside their current role. For example, they wanted training to administer injections and to check blood pressure, two tasks community members expect CHWs to be able to perform.

Training new CHWs has been more problematic. The length of training has not followed that prescribe by the policy. Health managers usually bring up a lack of budget as a reason for short trainings. The other reason mentioned by CHW trainers was the number of CHW trainees.

"*When we have one or two CHWs to train*, *it does not take that long to teach them everything*, *compared to having 10 or more CHWs in a class*". (CHW Trainer)

CHWs also blame some trainers who they say wish to go over the material as quickly as they can without caring if the CHWs actually learn it.

### Supervision

A Community Health Supervisor (CHS), a staff of a HF, supervises CHWs on a monthly basis, reviewing health activities such as the number of people they have visited, the number of patients they have referred to HF, the utilization of their drugs and supplies, and the registry of mother and neonatal morbidity and mortality. CHWs’ pictorial tally sheet will be recorded in another form called the Monthly Activity Record (MAR). CHSs will aggregate the MAR of every HP in a different form called Monthly Activity Aggregated Record (MAAR). These forms will be collected by the MoPH and will be entered into a large national database called the Health Management Information System (HMIS).

For CHSs, supervision is an opportunity to assess and improve the knowledge and skills of CHWs, and advise them on their service delivery, while for CHWs, supervision encourages them to work better, and is the time when they receive drugs and other supplies. As most CHSs are from a neighboring community, they are able to build a good rapport with the CHWs and the community. Their visit to the HPs gives credibility to the CHWs and, at times, they talk about the work of CHWs to the community and resolve issues between CHWs and the community. Most CHWs are satisfied with their supervisors, but they complain about a lack of drugs and supplies, which they know CHSs cannot do anything about. When there are such shortages, CHSs continue to supervise CHWs for their health promotion activities.

### Drugs and supplies

CHWs are provided with a box of drugs and supplies. It contains drugs for headache (Paracetamol®), pneumonia, diarrhoea and other infectious diseases (Co-trimoxazole), dehydration (ORS), and anemia (Ferric acid), multi-vitamin, eye ointment for eye infections, and family planning kit (contraceptive pills, condoms, and Depo-Provera®). Their supplies also include dressing materials such as bandage, hydrogen peroxide, gentian violet, scissor, forceps, and other basic health supplies. CHWs receive the drugs and supplies every three months.

Drugs are the most important element in establishing the role and subsequent effectiveness of CHWs. Village residents do not take CHWs seriously unless they have drugs that can be provided to them. Drugs give credibility to CHWs’ services, improve their referrals, and make their health advice more attractive and acceptable. Drugs from public health services are more effective than those bought from the local markets, which are mostly of low quality or falsified, imported from black markets of China and India.

"*The community learns about the arrival of drugs*, *the moment it reaches the health post. And then there is a crowd of villagers arriving with their pains and sicknesses. They often diagnose their own illness*, *and ask for a particular medication they know about*, *for example*, *the round white tablet in a transparent plastic bag*, *or the other white tablet in a cover*". (Female CHW)

### Gender dimensions

The CHW program is focused on maternal and child health, which makes female CHWs more effective in delivering the services. Almost all CHSs and health managers stated that women were better at CHW services for (1) the focus of the program on maternal and child health, (2) women’s availability and keenness for the CHW program, and (3) women’s ‘natural’ caring proclivities. Women’s interest in participating in the CHW program derives from the advantages it provides, especially for rural women. First, it gives them an opportunity to learn something. Most female CHWs take pride in learning about health matters, and that knowledge improves their social status.

"*I get to increase my awareness about pregnancy issues*, *and health matters*, *and also serve the people*". (Female CHW)

"*The people will not let me go now*… *some of them call me* ‘*respected doc*’". (Female CHW)

Second, their job as a CHW ensures them mobility. Their responsibility requires them to go to the houses of other villagers and promote healthy lifestyle and encourage women vaccination and antenatal, postnatal and delivery care. Typically, women need permission from their husband or a male head of the family to go out of their homes. The freedom to go out of their home to help others whenever they want to is experienced by CHWs as an empowering opportunity. Third, no matter how powerless these women may be in their society, they still hold the key to change in their home environment and in particular with their children. With increased knowledge, they demand clean water from their husband, require more water for cleaning purposes, want safer latrines from their husband, and encourage healthy behavior within their home.

Although our finding suggest that a majority of active CHWs are women, very few women are CHSs and even fewer women are found in managerial or policy making positions. This finding can be explained by the CHS job requiring extensive travel between villages, something with which most Afghan women are not socially comfortable, and which is difficult for women to negotiate. Although the Afghan minister of public health is a woman, all the deputy ministers and top decision-makers at the MoPH are men. Apart from gender differences embedded in the culture, this can also be explained by lower female secondary education in Afghanistan.

Despite the focus on maternal and child health, gender roles in the CHW program are not distinguished in the BPHS policy. It is the implementing organizations that take into account the gender sensitivities of the communities. According to the BPHS policy, there should be one female and one male CHW at every HP. In reality, the two CHWs at a HP could be a male and a female, or two females, or two males. Such arrangements depend on the culture of the area, availability of men and women for the position, or the attitude of the implementing organization towards gender issues. In the central government database, around half of the registered CHWs are male, but fieldwork for this study found that more female CHWs were active on the ground compared to their male counterparts. Gender dynamics were observed in the following aspects of the CHW program.

### Tasks

Although the BPHS policy states that all CHWs have similar tasks, the work of male and female CHWs are generally divided by gender roles in the community. Male CHWs have taken on environmental health services, raising awareness in public gatherings, treating common disease and injuries of boys and men, and facilitating transportation for patients to go to health facilities. Female CHWs are focused on maternal & child health and reproductive health of women such as family planning, vaccination of girls and pregnant women, antenatal care, delivery at a healthy facility, postnatal care, and home hygiene.

### Recruitment

It is preferred to have a male and a female CHW in a HP. It is preferred that male and female CHWs be *Mahram* (relatives of opposite gender allowed by Islamic law to interact). The permission of the male head of the family for a female to become a CHW is necessary. There have been many cases where single female CHWs stopped working after they got married.

### Training

The content of the training is similar for both male and female CHWs. The gender of CHW trainers depends on the attitude of the population. In central Bamyan, there is one male trainer for both male and female CHWs; however, in Kabul, Balkh and Parwan, female CHWs have female trainers and male CHWs have male trainers. At the community level, CHWs, both male and female, were satisfied with the teaching styles of both male and female CHW-trainers.

### Supervision

A single supervisor, either male or female, supervises both male and female CHWs; however, most CHSs are men, because men have the freedom to move and visit all the health posts in near and far villages covered by the health facility. Male CHSs build trusting relationships with the family of female CHWs in order to supervise and support them. They are mostly from a neighboring village that makes the rapport building easy.

"*We are like brother and sister*, *and I know all her family*". (Male CHS)

"*He is like family*, *he lives in Naal Shiran Balna*, *we do not have problem*". (Female CHW)

### Facilitators of the CHW program

#### Volunteerism

"[*I*] *want our community to prosper*, *if people appreciate our work*, *that*’*s better*". (Male CHW)

The successful recruitment of more than 20,000 volunteers for a government that is dependent on international donors is the biggest facilitator of the CHW program. CHWs are driven by several, intertwined motives for working as volunteers. CHWs report that they want to serve their community and to help their people. Most CHWs are called the village doctors, and they have the authority to distribute contraceptives, treat common childhood disease, provide counseling or refer patients to HFs. CHWs also seek to gain knowledge and skills which, they believe, will not only be advantageous to the community, but also to themselves and their families. CHWs, in particular those in their 20s or younger, hope that working as volunteer CHW is a step to further training opportunities to become a midwife, nurse, or even medical doctor, or a step to proper employment as a CHS.

"*Yes*, *this is a job for me*, *and if I stop doing it*, *it will be cheating*, *because we have committed ourselves to serve the community*, *although we do not get paid*". (Male CHW)

Although CHWs in Afghanistan are volunteers, they do receive monetary incentives such as travel expenses, lunch money, and educational stipends, and non-monetary incentives such as stationary for a community map, toothbrush and toothpaste, and hand soap. Monetary and non-monetary incentives are important but not deterministic in the decision to become a CHW.

#### Community involvement

Community involvement is another major program facilitator. The community nominates their trusted members to become CHWs. They form a village health council to discuss and resolve village health problems, and report it through the CHW and their representatives to the HF. They also form a Facility Health Council (FHC), in which they discuss and resolve their problems with staff of the HF.

"*When there was plan to build a health facility*, *there was not a proper place for it*, *the community identified a good piece of land close to everyone and gave it for the clinic*". (Male CHS)

These different ways of involvement give a sense of ownership over health services to the community, which in turn facilitates the CHW program. The village and facility health councils, for example, encourage community cooperation with the CHWs. Without the support of the community councils, CHWs could not even talk about health issues that used to be a taboo such as contraception, institutional delivery, ANC and PNC.

"*Sometimes*, *we ask community members to tell our messages through the mosque*, *so that everyone would listen*, *and they do it*". (Female CHW)

In most places, the community members have been helping CHWs with the transportation of pregnant women or other critical patients to HFs.

The FHC, in which representatives of all the communities served by the HF come together at the health facility, helps to keep otherwise isolated communities together, creating a sense of mutuality between the communities in good or in bad times. For example, when there is a lack of drugs, they know that all the communities have the same problem, and they get to decide whether to raise their voices collectively, or to accept the disservice without protest.

### Challenges

#### Retention of CHWs

"*They are volunteers*, *we cannot force them*, *if we put pressure on them*, *they just ask us to take the drug and supplies kit out of their house*". (Male CHS)

The volunteerism dimension of the CHW program can also be a challenge. CHWs volunteer for as long as they have the time and desire. Whenever they have better opportunities, they are likely to leave their volunteer work or give less time and attention to the community health services. If CHWs are asked to do more or better, they give up.

"*The terms of reference of CHWs are more than that of the minister of public health*, *if we expect them to actually do the work*, *we need to pay them*". (Policy Maker)

The volume of the work assigned to volunteers can also lead to dropouts. Most unmarried girls who became CHWs left that role after they got married, partly because, as a wife, they have tasks at home. The dropout rate estimated by the MoPH is only 5%, but the dropout rate on the ground appears to be much higher. An estimate from Bamyan province shows that that the dropout rate could be up to 10 percent.

To tackle the high dropout, the implementing organization has designed a ‘replacing CHW scheme’, according to which CHWs who drop out find and train replacements to take on the dropouts’ duties. It is not an official procedure, and there is no regular budget for it, and as already noted, replacement CHWs have never got the full basic training.

*A lot of our CHWs are replaced. This year almost 40 to 50 CHWs were replaced. Some of them went to teacher training colleges*, *or universities*, *or the rest of them got busy with their own works. They are simply replaced with another person from the village*. (CHW Trainer)

Basic six-month training for CHWs who are recruited to replace a dropout is almost non-existent. Generally, there is no budget for training a replacement CHW. The process is to file a notice about the dropout to the MoPH and request a budget to train new CHWs, a process that can take several months to a year before new training funds are obtained. Moreover, once the community is registered as ‘without CHW or a HP’, it will lose its drug and contraceptive supply and emergency transportation privilege. To avoid being out of the system for months, the community nominates another member who gets informal training from the previous CHW and the CHS, and immediately begins work as a CHW. In these cases, the name of the originally trained CHW remains in the database of the organization and the MoPH, but the CHS and the community know who is the actual CHW. The replacement CHW does not have any certificate of training, and will not receive an ID card as a CHW. If CHWs are considered to be the invisible army of primary care providers, the replacement-CHWs are the hidden invisibles. Six of the 25 CHWs interviewed for this study had not received the basic training, and they had been working between 1 to 6 years at the time of the interview.

#### Community challenges

Community involvement in recruitment does not imply the democratic participation of the community in the selection. Normally, the traditional chief of the village has the final word in the selection process. In some cases, chieftains have nominated their children, siblings or relatives to become CHWs. Chieftains have always had a room for community gatherings, which they then make into a HP.

CHWs are considered a bridge between the community and the health system, but they also take the blame from community members for the failures or weaknesses of both sides. When health services are not optimal, community members suspect CHWs are self-interested individuals who might have taken advantage of the resources provided to them. When there is a lack of drugs, CHWs are the first to be blamed for selling them at the local market or giving them to their relatives or friends or hiding them for themselves.

"*We are like the cursed*, *in the clinic they think we do not work*, *in the village people think we take the drugs for ourselves or sell it*, *they even blame us that we take dollars*". (Male CHW)

"*They say who works for free in these times*!?" (Male CHW)

Both of these suspicions are not based on evidence. The drugs CHWs receive, for example, cannot be sold in the market, as medications in ‘the transparent plastic bags’ are not for sale, a widely known fact.

CHWs also take the blame from the health system for not being able to change people’s unhealthy attitudes and behaviors. One of the common practices across Afghanistan, and probably in most developing countries, is not to seek medical care until the illness becomes life threatening.

"*They only come to us after they failed trying everything else*, *like Yunani medicine*, *prayers*, *keeping warm and even market medicines*, *and we cannot do anything but refer them to the clinic*". (Male CHW)

Due to the culture of self-care, western medicine is also often self-administered. Self-medication, which is particularly harmful for pregnant women, was observed to be prevalent. Another reason for self-medication could be the lack of physicians in rural Afghanistan. Overall, these socio-cultural practices represent a challenge for the CHW program, leading to underutilization of available services.

#### Health system challenges

Recognition and identification of CHWs by the health system, and appropriate drug supply and support for their work, are vital to their effectiveness.

### Recognition and identification

"*Once*, *I took a pregnant woman* [*who was*] *bleeding to a different clinic for delivery*, *but the clinic did not recognize me*, *so my patient and I had to wait so long until the baby was born* [*without medical staff assistance*], *and died later*". (Female CHW)

At the health facility, CHWs are considered *volunteer* community members who work for the sake of God and the service of their people, and are not a staff of the HF. The term volunteer connotes low-grade worker, compared to paid HF staff. A CHW in a village will stay a CHW in his/her village all his/her life. If they move to another village, they will no longer be a CHW because of the long, bureaucratic recruitment process. Although CHWs receive a certificate for their training that identifies them as CHWs in a hospital, their ill-treatment in a health facility damages CHWs’ credibility in the community and confidence in themselves.

### Drug and supply support

"*A CHW without drugs & supplies is like a soldier without his arms*". (Male CHS)

"*CHW loses trust*, *loses face* [*in the community*] *due to lack of drugs in the facility too*". (Policy Maker)

There is a general shortage of drugs for the CHWs and in the health facilities across the country. CHWs are provided with drugs and supplies every three months, but this supply usually lasts only for the first month. Because people are aware of this shortage, at the time of drug supply they rush to CHWs making up symptoms to receive drugs, and keep these for the time when they actually get ill. This high turn-out of patients during the early days of drug supply adds to the problem of shortages. Health Facilities suffer from the same problem. When drugless CHWs send ‘real’ patients (those needing treatment) to health facilities that have no drugs, patients and their families lose trust in the CHW, the health facility, and the whole service.

### Procurement and financing

"*If you want to buy a piece of paper*, *you need to waste ten of them first*". (Health Manager)

In Parwan Province, where the government implements the program, CHWs had not had drugs for almost nine months prior to the data collection. Bureaucracy in the government system is blamed for the long delay in drug supply. This was the one major difference in service provision between government-provided services, and NGO-provided services. NGOs, both national and international, make sure CHWs have their drugs at any cost and through any channel. The government department has to follow the slow bureaucratic procurement procedure to get the drugs.

The slow bureaucratic system within the government also affects the CHW program at the community level by interrupting their trip allowance and preventing CHW-trainers from providing basic training for replacement CHWs.

"*Our trip allowance for the last year has not arrived yet*, *how would they expect us to go to clinic again*?" (Male CHW)

It takes several months to a year for the annual budget to be approved first, then go through various departments, and eventually get to the provincial department, and then to health facilities. A lack of budget leads to no-payment to CHWs until the budget eventually arrives, and no training for replacement CHWs.

## Discussion

The BPHS program in Afghanistan follows the basic concepts of the Alma-Ata declaration on primary health care: ‘health as a fundamental human right’ and ‘equality in the health status of the people’ [15, 20]. The CHW program, a component of the BPHS, involves individuals and communities to take charge of health service provision in their community [15]. In some areas, rural development councils informally collaborate with village health councils to promote sanitation, safe water provision, and environmental health. However, at the policy level, this collaboration has yet to involve other sectors in the improvement of health-related services. An agreement between ministries of public health, education and rural development could increase community collaboration.

The CHW program, as a part of the BPHS, is an alternative to no organized public health care services in rural Afghanistan. The CHW program (20,000+ CHWs) has increased access to the primary health care services in rural Afghanistan [15, 16]. Increase in access to primary health care has been shown in other countries with national CHW programs, such as Ethiopia (30,000 Health Extension Workers) [5], Iran (91,000 Behvarz) [8], Pakistan (100,000 Lady Health Workers) [6], and Brazil (250,000 Community Health Agents) [9].

The design of the Afghan CHW program in regards to selection and recruitment from the community, and supervision and support by the health system, has been tested in other countries as well. CHWs selected and recruited from local communities improved health awareness and outcomes in low and middle-income countries [21–23]. Frequent supervision and adequate drug supply and support are crucial for the success of the program and the motivation of CHWs [24, 25]. In addition to those effects, in fragile states, countries in conflict, and post-conflict countries, CHWs help state legitimacy by being a representative of state-related health organization in the village, and protect health care providers and facilities through the involvement of community [26].

Gender roles were not explicitly taken into account at the BPHS policy level, and were strongly influenced in practice by social customs. Other countries with a similar cultural context as Afghanistan are neighboring Iran and Pakistan. Iran’s Behvarz program did not have any gender-related policies, and thus tasks were divided by gender roles in the society with female Behvarz providing services to women, and male Behvarz taking on tasks outside the household i.e. environmental health, following up communicable disease, and similar activities. [8]. Pakistan’s CHW program [Ladies Health Worker], on the other hand, is specifically designed to be by women for women and children [6]. However, even when health services are specifically focused on women and children, the involvement of women in the provision of community-level services is only visible at the volunteer level. There are few women supervisors, trainers, managers, and policymakers (Table 2).

Female CHWs tend to accomplish their roles as health providers better than their male counterparts. Male CHWs act as a complement to their female colleagues, facilitating transportation for female patients and CHWs, accompanying female CHWs outside the house and to the health facility, and undertaking some environmental health tasks. The relative lack of active male CHWs in communities was observed to be associated with poorer levels of community sanitation, provision of safe water, and a male support system. A contribution to gender equity in the health system in Afghanistan would mean advancing female and male CHWs through a potential career path in health as supervisors, trainers, and managers, which, as a result, could lead to greater health improvements and retention of CHWs in the community.

The CHW program in Afghanistan is also changing the dynamics of gender in the society, and empowers women. In patriarchal societies, like Afghanistan, the family has authority over the reproductive rights of women.

"…*her mother*-*in*-*law came here* [*at the HPs*, *saying*], … *you are not allowed to give my daughter*-*in*-*law contraceptives*’… *she told the entire village that I had given contraceptives to her daughter*-*in*-*law secretly*… *that fight lasted two*-*three months*". (Female CHW)

In liberal notions of empowerment, control over fertility/reproduction was a major topic in the first and second wave of feminism in wealthy first world countries. Provision of contraception to women in Afghanistan gives them an opportunity to control their reproduction.

A systematic review on CHWs in LMICs has identified management and supervision of CHW program to be an important enabling factor for the sustainability of such programs [27]. In the case of Afghanistan, a lack of drug supply and delay in government funding to run the program can be a major impediment to its quality and sustainability. We provide further and detailed insight on the sustainability of the CHW program in Afghanistan in a separate article.

### Limitation

The study had some limitations. A sample of 16 health posts to represent a CHW program in a country of multiethnic and multilingual context is small. However, we have taken into account various ethnicities, gender differences, extreme and relative rural places, and different implementers. Second, Afghanistan is defined by war; the safety of the researcher and key informants was a key concern. The researcher [MN] did not go to insecure provinces; did not stay in villages overnight; and travelled only when it was safe. However, he had the advantage of being local, speaking the languages, and knowing the context well. Finally, for a gender-segregated society, it is difficult for a male researcher to interact with female participants. To address this, a female research assistant was hired.

## Conclusions

This case study of the decade-long, rural health workforce CHW program in Afghanistan suggests that they play an important role in post-conflict, developing countries, potentially contributing to health system strengthening. They provide basic services to majority rural population, record and report health information from the villages and provide it to health managers and policy makers for better decision-making, and engage the community in strengthening the health system.

The results of this study offer two immediate policy-relevant outcomes. First, Afghanistan is going through a major transition in 2014 (presidential elections, international troop withdrawal, and changing international commitment/aid to Afghanistan). This will impact all aspects of the government system, including health services, and especially in rural areas. The results of this paper will inform the national and international organizations involved in the Afghan health service about the status of the decade-long rural health workforce program, potentially contributing to its strengthening. Second, to our knowledge, this paper is the first to document Afghanistan’s national rural volunteer health workforce program, thus contributing to the larger knowledge base on the role of CHWs in post-conflict, developing countries.
